# Human face images from multiple perspectives with lighting from multiple directions with no occlusion, glasses and hat

**DOI:** 10.1016/j.dib.2018.12.060

**Published:** 2018-12-21

**Authors:** Collin Gros, Jeremy Straub

**Affiliations:** a Department of Computer Science, Texas Tech University, United States; b Department of Computer Science, North Dakota State University, United States

## Abstract

Facial and other human recognition techniques are being used for a growing number of applications, ranging from device security to surveillance video identification to forensics. Data sets are required to test recognitions algorithms. This data set facilitates the evaluation of the impact of multiple factors on algorithm performance. The data set includes images taken under five different lighting levels (which vary in light brightness and temperature), seven different lighting positions and five different subject positions. The data set includes data collected for all combinations of the foregoing three collection variables, for a total of 175 images per subject. In addition, sets of data under three different occlusion conditions (no occlusion, glasses and hat) have been collected. Each data set includes images taken under all lighting level, lighting position and subject position combinations, for a total of 525 images of each subject. The images are all taken in the same location with the same background and camera equipment.

**Specifications table**

**Table:** 

Subject area	*Computer science*
More specific subject area	*Image processing & facial recognition*
Type of data	*Visible light images*
How data was acquired	*Camera - Canon EOS Rebel T2i with an Ultrasonic EFS 60 mm lens*
Data format	*Raw*
Experimental factors	*No data pre-treatment was performed.*
Experimental features	*Imagery of the subjects’ head and shoulder areas. Subject position, light position, light brightness and light temperature were varied. Hat and glasses occlusions image sets are included.*
Data source location	*Fargo, North Dakota, USA*
Data accessibility	*Mendeley Data*
Related research article	

**Value of the data**

• Facilitates the testing of facial recognition algorithms and the comparison of performance between algorithms.
• Provides data to evaluate algorithm performance under different subject position, lighting position, lighting brightness and lighting temperature conditions.
• Includes non-occluded data set and data sets with hat and glasses occlusions.
• Occluded and non-occluded data includes all lighting position, lighting level and subject position collection conditions.
• Data taken in controlled environment with a consistent white background.

## 1 Data

Identifying subjects from imagery [1] is required for numerous applications ranging from identifying customers in a retail store [2], [3] to applicant screening [4] to access control [5] to use by law enforcement [6]. Facial data can also be used to determine subjects’ interest levels [7], emotion [7], gender [8], [9] and age [8]. Techniques which use neural networks [10] and two-dimensional and three-dimensional approaches [11] have been proposed, among numerous other studies. Techniques for dealing with common problems such as limited training data [12], distortion [13], [14], occlusion [13], [14], [15], [16] and differing facial expressions [17] have been considered.

When facial recognition is used for security [18], [19], [20], [21], such as to allow access to a computer or mobile device [22], start a vehicle [23] or confirm the holder of a passport or ATM or credit card is legitimate [19], [24], attackers may seek to impair system performance [25], [26] by creating false positives (that authenticate a user) or false negatives, which result in users disabling authentication features. Environmental factors, including lighting, and occlusion may have an impact in this area.

This data set provides data that can be used to validate the performance of facial recognition systems under multiple conditions. It includes imagery of subjects collected from multiple perspectives, under multiple lighting brightness and temperature levels and with multiple lighting angles. A total of 175 non-occluded images are included, per subject. It also includes data with these characteristics where the subject׳s face is partially occluded by a hat or a pair of glasses. Thus, in total, 525 images of each subject are included in the data set.

## 2 Experimental design, materials, and methods

This section discusses the experimental design and the equipment and methods used. First, the specific equipment used will be discussed. The illumination levels produced by the different lighting configurations are also measured and presented. Then, a description for the setup used for taking photos and measuring the different light settings is included. Finally, the data collection procedure is discussed.

### 2.1 Equipment

The data set is comprised of multiple images. A Canon EOS Rebel T2i with an Ultrasonic EFS 60 mm lens was used for this photography. The camera was set in aperture priority mode, the camera used an ISO of 800, an FSTOP of eight, AI focus, auto white balance, auto exposure bracketing at −1 and spot metering. The flash was disabled and the camera was in landscape mode and set to produce images with 5184 × 3456 resolution. The camera also had peripheral illumination correction and red-eye reduction enabled. The photos were taken in screen preview mode, allowing the photographer to easily verify the camera׳s focus and pointing and saved in the JPEG format.

Two Neewer LED500LRC lights were used as background lighting. One Yongnuo YN600L was used for lighting the subject. A Tekpower lumen meter was used for collecting the measurements of lighting levels presented herein. A projector screen was used as the backdrop for the photos.

### 2.2 Setup

**Fig. 1:**
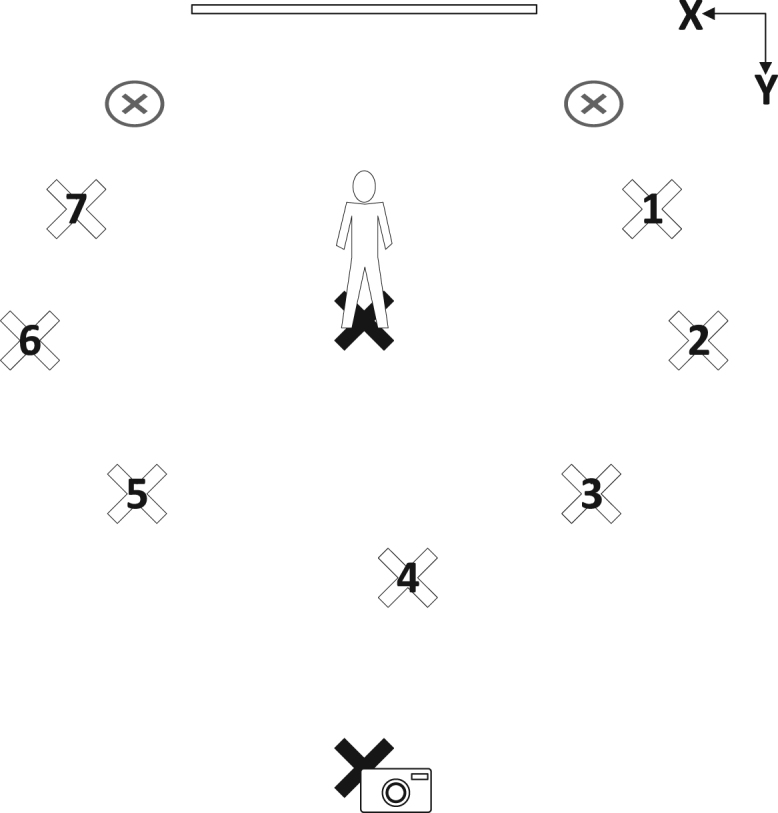
Subject, lighting and camera positions.

Seven different lighting angles were used. In each position, the light was pointed directly towards the subject. The camera was placed in a fixed position near the center lighting position. Fig. 1 depicts the positions of the light positions, the cameras, the projector screen backdrop and the subject. Note that this figure is not drawn to scale. It also notes the orientation of the coordinate system. Measurements of the precise locations of the camera, subject and lighting positions are provided in Table 1.

**Table 1:** Camera, lighting and subject positions (in inches).

**Location**	**X Coordinate**	**Y Coordinate**
Light 1	47.5	65
Light 2	44	87.5
Light 3	61	115
Light 4	84.5	127.5
Light 5	124	116.5
Light 6	198	91
Light 7	149	66
Background 1	43.5	50.5
Background 2	129	47
Camera	97	150
Subject	96.5	63.5

### 2.3 Lighting

**Fig. 2:**
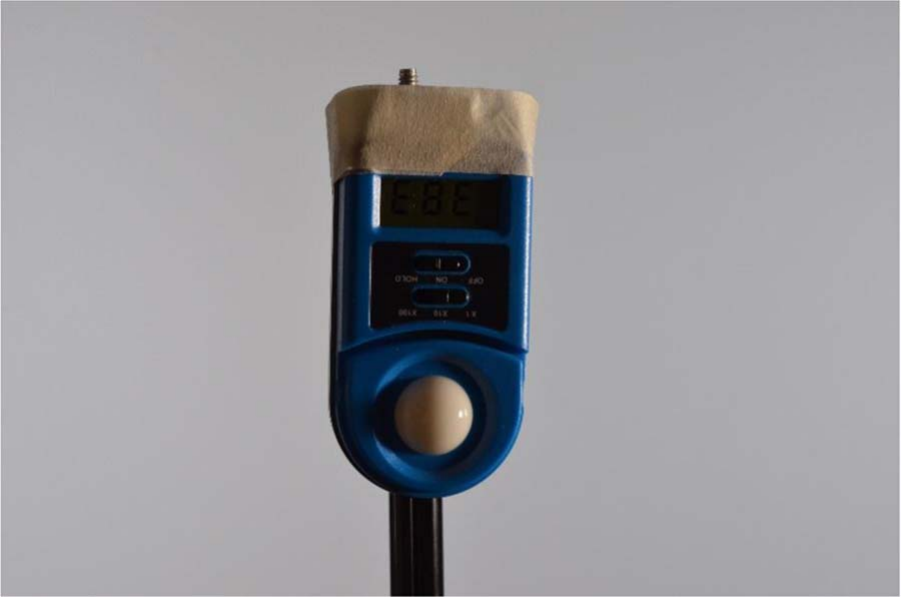
Lumen meter used for light measurements.

**Fig. 3:**
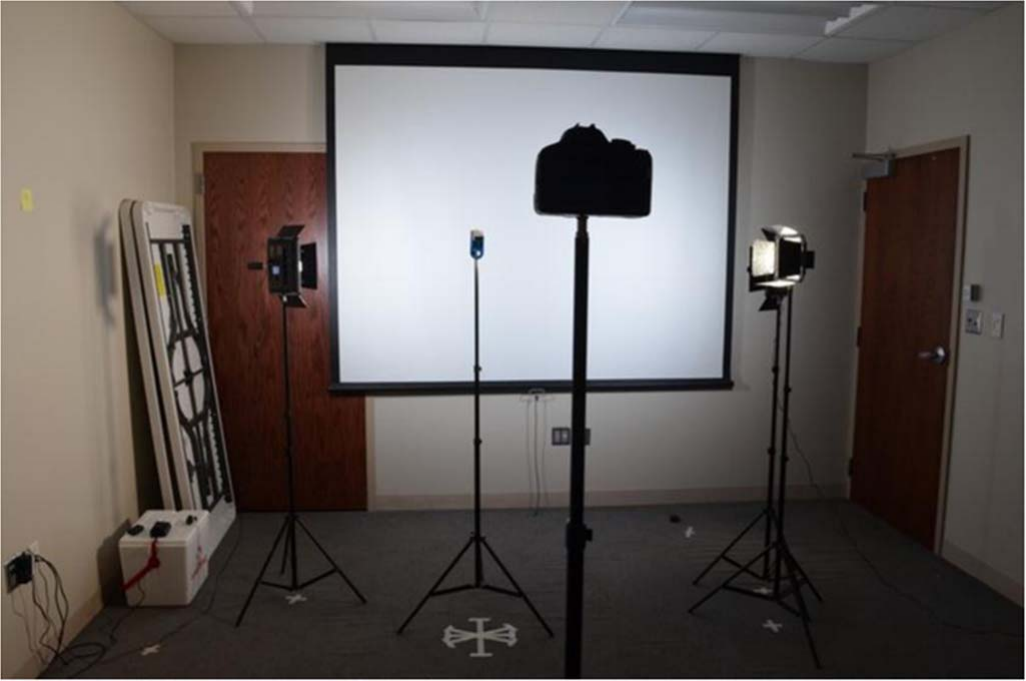
Photography setup showing lumen meter collecting data.

Data was collected under multiple lighting conditions. The Yongnuo YN600L LED light, which was used to illuminate the subject from multiple locations was varied between multiple settings: 60% brightness on warm (3200k), 60% brightness on cold (5500k), 10% brightness on warm (3200k) and 10% brightness on cold (5500k), 40% brightness on warm (3200k) and 40% on brightness on cold (5500k), and 70% brightness on warm (3200k) and 70% brightness on cold (5500k). For ease of reference, each of these settings configurations was given a name. These configurations and names are listed in Table 2. The brightness levels were measured from a lumen meter facing the camera, as shown in Fig. 2 and with its position shown in Fig. 3. Lumen readings were taken under all lighting levels and with the light in all positions. This data is presented in Table 3.

**Table 2:** Lighting configurations and their titles.

Configuration title	Settings
Warm	60% brightness on warm (3200k)
Cold	60% brightness on cold (5500k)
Low	10% brightness on warm (3200k) and 10% brightness on cold (5500k)
Medium	40% brightness on warm (3200k) and 40% on brightness on cold (5500k)
High	70% brightness on warm (3200k) and 70% brightness on cold (5500k)

**Table 3:** Lumen levels produced by multiple lighting positions and temperature and brightness levels.

	Angle 1	Angle 2	Angle 3	Angle 4	Angle 5	Angle 6	Angle 7
Warm	238	248	278	280	297	219	131
Cold	317	340	386	391	414	301	177
Low	129	137	154	155	164	122	76
Medium	380	403	458	492	489	357	210
High	610	648	735	745	788	572	332

### 2.4 Subjects and procedure

The subjects were males between the ages of 18 and 26. Several subjects had beards others had less pronounced facial hair.

**Fig. 4:**
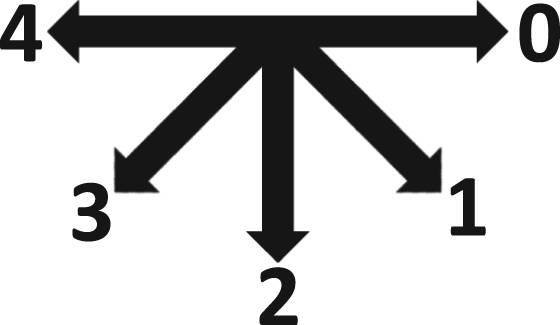
Orientation of subjects.

Each subject was instructed to face five different directions, with their head pointing forward relative to their body position. In each position, the subject was asked to remain still, while the subject illumination light was moved to each location, starting in position 1 (far right, facing the subject) and ending in position 7 (far left, facing the subject), light brightness and temperature levels were changed and photos were taken. The light movement and brightness and temperature adjustment procedure was repeated for each position. When each position׳s data collection was complete, the subject was asked to turn to the next position, until the subject reached the final position, which was 180 degrees from the direction they were first facing. A diagram of the positions that the subject was asked to face is presented in Fig. 4. Table 4 lists the lighting angle for each combination of subject orientation and lighting position. Across the different orientations and lighting configurations numerous lighting angles are created, facilitating system testing under numerous photography and lighting angles.

**Table 4:** Lighting angle (in degrees) for each subject orientation and light position.

		Subject orientation				
		0	1	2	3	4
Light position	1	2	17	92	137	182
	2	25	20	65	110	155
	3	55	10	35	80	125
	4	79	34	11	56	101
	5	117	72	27	18	63
	6	165	120	75	30	15
	7	183	138	93	48	3

**Fig. 5:**
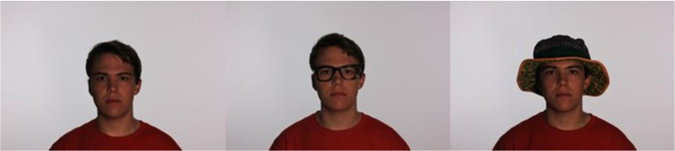
Photos of three different sets were taken for each subject.

Three sets of images were taken for each person. The first set consists of all possible lighting positions and temperature and brightness levels and subject positions without the subject having any facial occlusion. The second set features the subject wearing a set of glasses (without lenses, to avoid glare). The third set features the subject wearing a hat. Images under all subject position, lighting position and lighting temperature and brightness conditions were collected for both occlusion conditions, as well. Fig. 5 depicts a subject in each configuration.

**Fig. 6:**
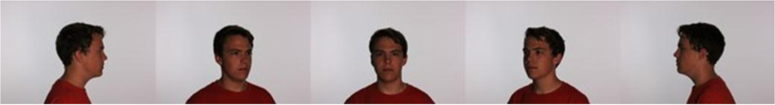
Photos of five different positions were taken for every set.

Subjects were asked to keep a neutral face and face the forward, relative to their body position. A subject, in each of the five different positions, is shown in Fig. 6.

**Fig. 7:**
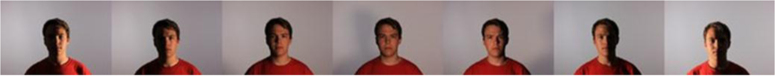
Photos of seven different lighting angles were taken for every position.

While the subject stood in each position, the Yongnuo YN600L LED subject illumination light was moved to each position depicted in Fig. 1 and turned to face the subject. Fig. 7 shows a subject illuminated from each angle.

**Fig. 8:**
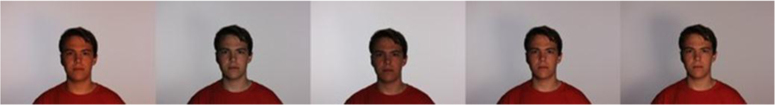
Photos of five different lighting settings were taken at every angle. From left to right: warm, cold, low, medium and high.

In each position, the brightness and temperature settings of the Yongnuo YN600L subject illumination light were changed. Fig. 8 depicts a subject photographed in a single position with a single lighting position under each brightness and temperature setting.

**Fig. 9:**
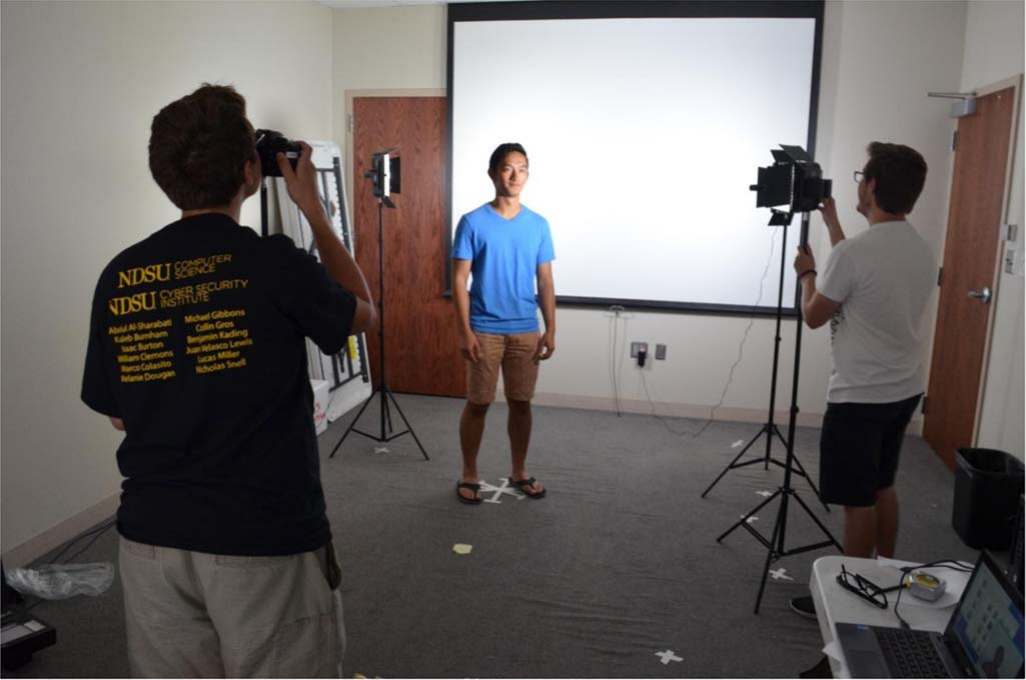
Subject being photographed.

The overall collection setup is shown in Fig. 9. After each set of photos were taken, they were reviewed to ensure that the subject did not blink or show emotion. If the subject did either, the relevant image was deleted and taken again. The images were also reviewed on a higher-resolution (compared to the camera screen) screen to ensure their quality and focus and images were retaken, if any problem was detected.

## 3 Data collection controls

A key goal in the collection of data was to control as many factors as possible outside of those intentionally varied. The data collection process used a consistent script and procedure. The images were taken entirely in the same location, using the same lighting configurations. Doors were closed at the time and no other (non-controlled) lighting was present. Markers were placed on the floor (as shown in Fig. 8, under the subject being photographed) and on the walls to direct the subject as to what direction to face and what direction to look. Images were manually checked after each subset were taken to identify any significant anomalies.

The shirt that the subject was wearing was not controlled and subjects were photographed in whatever shirt they arrived in. Subjects were not asked to alter their appearance, beyond wearing the hat and glasses at the appropriate time (and not wearing any other hat or glasses at other times). The glasses and hat were provided and were the same between subjects. There was minor fluctuation in the exact position of the subject. Beyond the subjects’ attire, position and facial state, all other aspects of data collection were controlled.
